# Biomarker as a Research Tool in Linking Exposure to Air Particles and Respiratory Health

**DOI:** 10.1155/2015/962853

**Published:** 2015-04-23

**Authors:** Nur Faseeha Suhaimi, Juliana Jalaludin

**Affiliations:** Department of Environmental and Occupational Health, Faculty of Medicine and Health Sciences, Universiti Putra Malaysia, 43400 Serdang, Selangor, Malaysia

## Abstract

Some of the environmental toxicants from air pollution include particulate matter (PM_10_), fine particulate matter (PM_2.5_), and ultrafine particles (UFP). Both short- and long-term exposure could result in various degrees of respiratory health outcomes among exposed persons, which rely on the individuals' health status. *Methods*. In this paper, we highlight a review of the studies that have used biomarkers to understand the association between air particles exposure and the development of respiratory problems resulting from the damage in the respiratory system. Data from previous epidemiological studies relevant to the application of biomarkers in respiratory system damage reported from exposure to air particles are also summarized. *Results*. Based on these analyses, the findings agree with the hypothesis that biomarkers are relevant in linking harmful air particles concentrations to increased respiratory health effects. Biomarkers are used in epidemiological studies to provide an understanding of the mechanisms that follow airborne particles exposure in the airway. However, application of biomarkers in epidemiological studies of health effects caused by air particles in both environmental and occupational health is inchoate. *Conclusion*. Biomarkers unravel the complexity of the connection between exposure to air particles and respiratory health.

## 1. Introduction

Air pollution is a major threat associated with the environment around the world because everyone needs to breathe to survive. We breathe in almost anything that is in the air, which includes particles from air pollutants. Depending on the size of the particles, some of the particles could deposit anywhere along the respiratory tract or, worse, penetrate deep into the gas exchange region [1].

PM_10_ is particulate matter with an aerodynamic diameter of 10 micrometers or less; these particles are more likely to go past the nose and the mouth. PM_2.5_-PM_10_ is referred to as the coarse fraction. PM_2.5_ is particulate matter with an aerodynamic diameter of 2.5 micrometers or less; these particles are more likely able to reach the deeper parts of the respiratory tract. It is known as the fine fraction. It can enter the respiratory tract and penetrate deep into the lung because of its fine size [2]. Ultrafine particle (UFP) is air particle less than 0.1 micrometers. When it is compared with larger particles on a mass basis, UFP has a higher predicted pulmonary deposition, greater potential to induce pulmonary inflammation, larger surface area, and enhanced oxidant capacity [3].

Inflammation is the first responder of immune response to infection or injury in the body. Inflammation is detected by an increased concentration of biomarkers in the human body. This inflammatory action is meant to take place in a short time. When the response proceeds for a longer period, it is probable to damage the body. Unfortunately, the defined mechanisms underlying the association between air particles and an increased risk of respiratory symptoms are still unclear. Inflammatory response is connoted as a biologic tool that links air particles with health effects [4].

It is crucial to establish a few factors in determining the possible mechanisms to relate concentration of inhaled particles with any toxicological effects. Those factors include the surface characteristics and electrostatic charge of the particles, their size, their internal structure, their site of deposition, their physicochemical structure, and their fate after retention within lung, whether they carry any chemicals in solid, liquid, or gas forms, and whether there is evidence that they trigger any toxicological response [5]. Upper respiratory tract mostly provides physical barrier against harmful insults, while lower respiratory tract mostly provides biological defense against harmful substances.

There are various components of air, some of which are air pollutants. These pollutants are in the form of free radicals in their individual forms or are able to generate free radical reactions. As a result, exposure to mixture of air pollutants in the atmosphere is building oxidative stress within the lung, which later triggers inflammatory responses. Oxidative stress is an imbalance between reactive oxygen species (ROS) and the antioxidant defense mechanisms. This is an important cause of oxidative injury. The oxidation of these biomolecules is usually accompanied by inflammatory responses, followed by human diseases associated with free radical formation. Exposure to environmental toxicants is associated with the generation of free radicals. In animal studies, vehicular traffic exposure is shown to exacerbate allergic inflammation in already-sensitized rats [6].

Many studies have found that exposure to PM_10_ promotes inflammation in the lung, which is related to a systemic inflammatory response [7]. Inhalation of air particles is thought to affect the heart and the lungs. The lungs are affected by the inhalation of air particles, which are brought deep into the lungs and are deposited in the alveolar sacs. Particles are deposited into the epithelium of the lungs and are thought to be capable of moving into interstitial spaces between cells. The particles then induce inflammatory responses [8].

Some of the significant health effects of concern from the exposure to particulate matter include aggravated asthma, chronic bronchitis, chronic cough, breathing difficulty, and others. These health effects could become serious and can cause discomfort to the affected individuals, which may also lead to absenteeism from work or school, frequent emergency room visits, and hospital admissions for respiratory problems or, worse, morbidity. The health effects are mostly profound among populations who are at risk, which include children, the elderly, and individuals with respiratory illnesses.

Biomarkers to be used in studying the exposure level of air particles must be valid and relevant. Environmental exposures are usually irregular in their magnitudes over time, thus posing a challenge to measure a specific biomarker if the time of the measurement changes. It is always assumed that biomarkers levels measured are at a constant level in the body and biomarkers are most responsive to the latest detrimental exposures [9]. It is also important for the biomarkers to have sufficient sensitivity to be detected in giving information to link environmental exposure and health outcomes [4].

It is improbable that there is a single biomarker to relate health outcomes with a particular exposure because a group of biomarkers are needed to imitate both short- and long-term exposures. Multiple biomarkers represent various elements of disease pathways. Therefore, there is no single biomarker that can ideally suit environmental health study needs. Moreover, when biomarkers are used to evaluate exposures and outcomes, more accurate evaluations are possible than in studies using monitoring data only [10].

The understanding and availability of information on this matter have been improved as time flies. Since the last two decades, biomarkers have been utilized in environmental health fields to improve exposure assessment and to learn the related diseases mechanisms. Biomarker is an entity in which organisms show the functionality of a biological organism in a variety of biological processes involved in life [4]. This review article is written to delve into the assumption that biomarkers are useful in linking harmful air particles levels to increased respiratory health effects. It will be useful for the researchers involved to view and compare the general inflammatory properties of selected biomarkers in one piece of literature.

## 2. Methods

This review was done to strengthen the view that biomarker is relevant to be used in understanding the health effects of respiratory system caused by exposure to air particles. The literature research was conducted by using online journal databases. The selection of published journals to be reviewed was made based on the following criteria:

- ambient or personal exposure to any of the air particles (PM_10_, PM_2.5_, or UFP);
- using biomarkers to diagnose the air particles exposure;
- health outcomes related to respiratory health;
- studies done to determine the association between exposure to outdoor or indoor air particles (PM_10_, PM_2.5_, and UFP) and respiratory health effects;
- journals published in English from 2003 to 2014.

Relevant studies were identified from scientific journals search engines, which were PubMed, ScienceDirect, Scopus, and Google Scholar. The following keywords were used: “PM_10_ biomarker,” “PM_2.5_ biomarker,” and “UFP biomarker.” Meanwhile, additional references for scientific journals related to the topic discussed in the Results and Discussion were obtained from the list of references attached at the end of the selected journals.

The keyword “PM_10_ biomarker” yielded 38 results when searched on PubMed, 443 results when searched on ScienceDirect, 36 results when searched on Scopus, and 7380 results when searched on Google Scholar.

The keyword “PM_2.5_ biomarker” yielded 77 results when searched on PubMed, 471 results when searched on ScienceDirect, 52 results when searched on Scopus, and 5810 results when searched on Google Scholar.

The keyword “UFP biomarker” yielded 16 results when searched on PubMed, 60 results when searched on ScienceDirect, 7 results when searched on Scopus, and 1260 results when searched on Google Scholar.

Search findings were excluded if their studies did not control potential confounders that might contribute to air particles exposure. Detailed findings of statistical data analysis and exposure assessment, for example, mean concentrations of air particles, were not included. A simple compilation of these reviewed journals is made easier by combining them in a table. Further interpretation of these journals, which include reported associations between concentrations of air particles and concentrations of biomarkers, was highlighted.

## 3. Results and Discussion

Studies of biomarkers as tools in revealing exposure to air pollutants had been found from all over the world as listed in Table 1. For the purpose of this review that is to provide fundamental information regarding the application of biomarkers in epidemiological studies of assessments of air particles exposure, we shortlisted 7 journals meeting our search criteria. The information obtained from the journals is summarized in Table 1.

A cross-sectional study by Vattanasit et al. [11] in Thailand showed induction of oxidative DNA damage and inflammation caused by PM_2.5_. They studied proinflammatory cytokine (IL-8), lung secretory protein (CC16), and 8-OHdG as biomarkers to investigate personal PM_2.5_ exposure of 51 healthy residents aged 18–58 years. To minimize the potential confounders, the subjects were selected among those living or working within 500 m from the main road of study sites. There were 31.4% male and 68.6% female subjects recruited. They carried out two studies: human and* in vitro* study. In the human study, the concentration of 8-OHdG in leukocytes was significantly associated with the individual exposure to PM_2.5_. Conversely, no significant correlation was observed between PM_2.5_ exposure and expression of IL-8, which was not what they had expected because the* in vitro* study conducted in the same study showed a positive expression of IL-8. Meanwhile, the correlation between exposure to PM_2.5_ and serum CC16 expression was inconclusive. No respiratory symptoms were reported.

Dobreva et al. [12] did a cross-sectional comparative study showing PM_2.5_ and PM_10_, which interrupted the balance between anti-inflammatory IL-10 and proinflammatory TNF-*α* in Bulgaria. They studied changes in the plasma level of TNF-*α*, IL-6, IL-12p40, and IL-10 to investigate 18-year-old adolescents continuously exposed to different degrees of ambient air pollution in three cities, which were Stara Zagora, highly industrialized with intense traffic; Kazanlak, moderately industrialized with intensive traffic; and Chirpan, rural town with agricultural plants. A total of 40 adolescents (55% female; 45% male) from Stara Zagora, 37 adolescents (54% female; 46% male) from Kazanlak, and 37 adolescents (51% female; 49% male) were involved. To minimize the potential confounders, the samples of ambient PM were taken in October, a month without pollens or other plant allergens in the air. The concentration of TNF-*α* was found to be the lowest among adolescents living in Stara Zagora compared to the other two areas. This was in accordance with the highest concentration of IL-10 found in adolescents living in Stara Zagora. On the other hand, the concentration of IL-6 and IL-12p40 had no significant changes among the adolescents from the three different areas. No respiratory symptoms were reported.

In a cross-sectional comparative study of 430 healthy schoolchildren aged 8 and 10 years in Malaysia, Nazariah et al. [13] reported that a positive association was found between indoor concentration of PM_2.5_ and PM_10_ and the concentration of IL-6 among the schoolchildren. Indoor air measurement was taken in the children's homes. There was no specific gender of respondents mentioned by the authors. Potential confounders were minimized by selecting children who lived within 5 km radius from their schools. The range of indoor levels of both PM_2.5_ and PM_10_ in urban area homes was higher than the indoor levels found in rural area homes. Those living in urban areas were indicated to significantly express a higher concentration of IL-6. Besides that, they demonstrated that both PM_2.5_ and PM_10_ were significantly correlated with all respiratory symptoms except chest tightness.

Elango et al. [14] highlighted the association between personal exposure to air particles, inflammation, and oxidative stress in photocopy centers in India. They carried out a cross-sectional comparative study involving 81 photocopier operators (79% male; 21% female) and 43 healthy controls (72% male; 28% female) aged between 20 and 60 years from the same geographical area. The controls include shopkeepers, housewives, and clerks. Interference of biomass fuel smoke was eliminated by choosing subjects who did not use that as household cooking fuel. Cigarette smoking was a confounder, but the lung function data of smokers were not significantly different from the nonsmokers. A high prevalence of respiratory problems such as nasal blockage, breathing troubles, and excessive sputum production was found among the photocopier workers compared to the control subjects. Levels of serum TBARS, plasma ICAM-1, LTB_4_, plasma ECP, and plasma IL-8 were significantly increased in the photocopier operators compared to the controls. Conversely, the level of serum FRAC was significantly decreased among the photocopier workers in comparison to the nonexposed group. CRP and CC16 showed no significant differences between the compared groups and thus no correlation among the groups. The authors demonstrated that air particles might contribute to respiratory symptoms.

Kavitha et al. [15] demonstrated associations of personal exposure to indoor air particles with IL-6 and TNF-*α* in Klang Valley, Malaysia, with its highly developed road network. They carried out a cross-sectional comparative study among 20- to 55-year-old nonsmoking and healthy bus drivers and administrative staffs, 62 respondents for each group. The authors mentioned no specific gender of respondents. The exposure measurement was taken within driving zone for the bus drivers when the buses were in motion and within working zone for the comparative group. The bus drivers had a significantly higher mean level of TNF-*α* and IL-6 concentration compared to the comparative group. A statistically significant positive association was observed between air particles concentrations and IL-6 concentration. A positive correlation was also observed between air particles concentrations and TNF-*α* concentration. No respiratory symptoms were reported.

In a crossover study involving 60 asthmatic patients in the United Kingdom by Zhang et al. [16], they used eNO, pH of EBC, blood TBARS, and sputum IL-8 and MPO in investigating the short-term exposure level of PM_2.5_ and UFP to the subjects. They were nonsmokers aged between 18 and 55 years with almost the same number of male and female subjects. They were also not involved in occupations entailing high exposure to air particles and did not reside or work near the exposure site. The study consisted of one exposure session and one control session with separation of at least 3 weeks. The exposure session was carried out near Oxford Street, a busy street in London. During the day when the study was carried out, only diesel-engine vehicles were allowed on the street. Meanwhile, the control study was carried out in Hyde Park, a large park with less traffic near Oxford Street. Pollen season was also avoided by carrying out sessions between November and April. The same instructions were given to all subjects to reduce bias. It was found that exposure to UFP was significantly associated with pH of EBC, sputum IL-8, and sputum MPO. Subjects with moderate asthma had lower pH of EBC compared with subjects with mild asthma. Concentrations of both sputum IL-8 and MPO were reported to be increased after the exposure session. In contrast, no significant changes were detected in blood TBARS and eNO after exposure to the air particles. No respiratory symptoms were reported.

In a panel study reported by Sørensen et al. [17] which involved 20- to 33-year-old students in Copenhagen, Denmark, 8-oxodG was used as a DNA damage biomarker to assess the personal exposure of the subjects to PM_2.5_. They were nonsmokers living and studying in the central region of that metropolitan city, with an even distribution of males and females. Indoor measurement was taken from instruments, which were placed in the students' backpacks; outdoor measurement was taken from a rooftop of a university campus building. They found that there was a positive association between air particles exposure and 8-oxodG concentrations in lymphocyte DNA. Frequent exposure to the air particles may result in an increased oxidative stress. To account for seasonal variation that might have an impact on the exposure levels of PM_2.5_, the measurements spanned 1 year and included one measurement for each season. No respiratory symptoms were reported.

As time flies, advancement in technology and modernization claim for better transportations modes and lifestyles. Routines are going at a fast pace especially in the urban and industrial areas. Reliance upon motor vehicles has resulted in increased atmospheric air particles, whereas industrial activities that have progressed a lot with bigger productions each year have also contributed to the large amount of air particles in the atmosphere.

There are speculations that air particles contribute to respiratory problems especially to vulnerable populations such as children. However, it remains controversial, as there are various findings about this matter. Exposure to air particles may result in respiratory health effects with various degrees depending on the concentration and duration of exposure. In toxicology tenet, inhaled dose is used in predicting the health risk, not the concentration of the exposed particles. Due to diverse compositions and concentrations of particulate matter, differences among studies may exist.

Compared to stationary monitoring stations, personal exposure assessment is deemed to be more strongly associated with an increase in inflammatory biomarkers. Numerous studies since the 1990s have shown that inhaled particles induce oxidative stress and inflammation, those of which are important mechanisms for health effects [18, 19]. These studies provide supportive evidence from different perspectives for applicability of biomarkers in epidemiologic research related to air particles exposure.

An inflammatory response is referring to the permeability of the alveolar-capillary barrier, which leads to serious fluid and blood cells effusing from the capillary side into the alveolar spaces [20]. Generally, this injury response process involves penetration of leukocyte into the injured area. When discussing respiratory system, lung's first response against the introduction of foreign substances takes place as neutrophils and alveolar macrophages infiltrate the lung. It is capable of handling familiar enemies; thus defenses are developed for the latter threat, not the former threat that is still new to the lung.

Biomarkers are effective to determine exposure to air particles. However, the individual outcomes of air particles in causing health effects are hard to be identified because each of them has a unique and complex mixture that comprises those tiny organic and inorganic compounds. Individuals with underlying inflammation in their respiratory tracts such as asthma and allergy are more vulnerable to the effects of particulates exposure. Sometimes, genes may play a role in enhancing inflammatory responses and developing respiratory health symptoms. Meanwhile, variations in traffic volume and weather conditions could also lead to variability of pollutants concentrations [16].

There are reasonable grounds for concern that air particles may have bad impacts on the respiratory health. High concentration of air particles is proven to either lead to respiratory diseases or have already caused respiratory symptoms among exposed subjects.

Nevertheless, the lack of established mechanisms of action has made it difficult to reach a general conclusion that air particles are causing both acute and chronic health effects, depending on the dose and length of exposure. Besides that, the presence of high concentrations of adsorbed chemicals on the surface of the particles deposited may have affected the toxicological response induced.

Even though certain aspects especially the mechanisms of inflammatory responses involved remain unanswered, there are already some well-documented and well-established findings regarding the adverse health effects from exposure to particulate air pollution. These well-documented findings have strengthened the idea of using biomarkers in studying the health effects associated with air particles exposure.

*Biomarkers of DNA Oxidative Damage.* The spectra of 8-oxodG produced as a result of DNA oxidative damage have been used in numerous studies to assess exposure of subjects to air particles. 8-OHdG, also known as 8-oxodG, is one of the major DNA modifications by ROS [11]. ROS can oxidise DNA molecules to form 8-OHdG that are readily excreted in biofluids such as urine [21]. The data by Vattanasit et al. [11] and Sørensen et al. [17] show that 8-OHdG was positively correlated with exposure levels of PM_2.5_, which are similar to findings from other studies [22, 23]. The increased formation of 8-OHdG signifies oxidative damage, which was probably caused by frequent exposure to air particles. Oxidative damage in cells then caused inflammatory responses to take place; thus the high expression of IL-6 was observed in the* in vitro* study by Vattanasit et al. [11].

Antioxidant works to offset the oxidative damage caused by ROS. Despite being a useful indicator to measure the antioxidant capacity of an organism, FRAC has not been widely used as a biomarker of oxidative stress due to exposure to air particles. This rapid and simple method to detect oxidative stress in serum is based on the reduction of ferric ions in the serum [24]. Elango et al. [14] found a low concentration of FRAC among the photocopier operators when compared to the healthy group. The low concentration exhibits low antioxidant capacity and, therefore, signifies the presence of oxidative stress due to long-term exposure to photocopiers emissions.

Another biomarker that detects oxidative stress is TBARS. These groups of low molecular weight chemicals are formed during the decomposition of lipid peroxidation products [25]. Oxidative stress is determined with an increase in lipid peroxidation. As to our knowledge, the usage of TBARS as a biomarker of inflammation due to exposure to air particles is not widely studied. Elango et al. [14] found a high concentration of TBARS among the photocopier operators when compared to the healthy group. The high concentration of TBARS is associated with increased oxidative stress [26–28] due to exposure to particulate pollutants from photocopiers emissions.

A decrease in exhaled breath condensate pH has been associated with increases in airway inflammation [29, 30]. Zhang et al. [16] demonstrated a positive association between exposures to UFP with pH of EBC. Meanwhile, in some other studies, increasing exposure to PM_10_ and PM_2.5_ was associated with acidity of EBC [31, 32]. This acidic pH can induce oxidative stress, thus promoting activities of proinflammatory agents. However, the unstable parameter of its acid-base property makes it a nonspecific biomarker.

*Biomarkers of Inflammation following Oxidative Damage.* Several studies have shown proinflammatory responses of IL-8, also known as CXCL8, in relation to exposure to air particles [33–35]. In view of this proinflammatory property of IL-8, it is expected that it would show positive expression towards exposure to air particles because production of IL-8 is implying defense mechanism to airborne particles. IL-8 has two specific actions on neutrophils: it is a potent chemotactic agent, and it activates neutrophils to degranulate and release oxidants [36]. Elango et al. [14] and Zhang et al. [16] observed a positive correlation between the air particles concentrations and IL-8 concentration. Such data have shown that increased levels of IL-8 to signify inflammation are present among the subjects. On the other hand, Vattanasit et al. [11] did not observe any significant correlation between PM_2.5_ exposure and expression of IL-8 for their human study, which may suggest that either the inflammatory activation in the airways was downregulated by some mechanism or the level of PM_2.5_ exposed was not high enough.

Previous studies have well addressed the proinflammatory behavior of IL-6 in cytotoxic effects [37] and its positive association with levels of macrophage ROS generation [38] and summer weather [39]. Meanwhile, some other studies portrayed the anti-inflammatory behavior of IL-6 with its regenerative function [40, 41]. These are proving the dual functions of IL-6 as both a proinflammatory cytokine and an anti-inflammatory cytokine. Sources of IL-6 include fibroblasts, macrophages, T lymphocytes, and monocytes [36]. Nazariah et al. [13] and Kavitha et al. [15] observed a positive correlation between the particulate matter concentrations and IL-6 concentration, which are similar to several studies [39, 42]. When the level of IL-6 was found to be elevated in the subjects, this has proven IL-6 for its proinflammatory role in the respiratory inflammation after the subjects were exposed to PM_2.5_ and PM_10_. Nevertheless, Dobreva et al. [12] did not detect any significant change in the levels of IL-6 in the subjects exposed to PM_10_ and PM_2.5_. The low levels of IL-6 expressed may be caused by the anti-inflammatory reaction of IL-6.

Although IL-12p40 has been associated with pathogenic inflammatory responses, there are meager studies done on the usage of IL-12p40 as a biomarker of inflammation due to exposure to air particles. The production of this proinflammatory heterodimer cytokine forms a link between innate resistance and adaptive immunity [43]. Dobreva et al. [12] did not detect any significant change in the concentrations of IL-12p40 in the subjects from the three different areas exposed to PM_10_ and PM_2.5_. This could probably be due to the low concentration of PM_10_ and PM_2.5_ exposed, which did not really trigger inflammatory responses in the subjects' bodies. Nevertheless, that is not the reason. Manipulation of inhibitory pathways could be limiting the inflammation; thus no significant changes were observed in concentrations of IL-12p40.

Proinflammatory responses to foreign particles are essential for host defense, but uncontrolled inflammation may result in a more serious health condition such as serious tissue damage or even death. Therefore, it is also important to have mechanisms limiting inflammation. Resolution of inflammation is characterized by IL-10 expression. This anti-inflammatory cytokine has been documented to mediate its inhibitory effects on cytokine production, thus reducing production of proinflammatory cytokines and chemokines, including IL-8, TNF-*α*, and IL-12 [44]. Dobreva et al. [12] had shown that the elevated concentration of IL-10 as the anti-inflammatory cytokine had suppressed the proinflammatory response of TNF-*α*. This is demonstrating the central role of IL-10 in regulating immune responses to maintain a good immune homeostasis.

A broad range of studies revealed the central role of TNF-*α* along with some other cytokines in inflammatory responses resulted from the inhalation of air particles either indirectly or directly [42, 45]. Its role is to eradicate foreign agents. High concentration of TNF-*α* is found to be detrimental as it may induce cell death, while normal or low levels of TNF-*α* are protective and safe and are believed to participate in normal lung function [46, 47]. Dobreva et al. [12] found a low level of TNF-*α* but a high level of IL-10 in adolescents living in the highly industrialized city. This was probably due to the regulatory function of TNF-*α*, apart from its normal proinflammatory property. This finding is indicative of an immune dysregulation due to long-term exposure to air particles. In contrast, Kavitha et al. [15] observed a positive correlation between the particulate matter concentrations and TNF-*α* concentration. The elevated concentration of TNF-*α* indicates that an inflammatory reaction is taking place from the formation of cellular oxidative stress.

Previous studies have linked air pollutants with elevated CRP levels in various populations of subjects, but the results have been varying [1, 48]. This protein is produced by the liver and is widely used as an indicator of systemic inflammation. Its expression increases in the presence of inflammation. Elango et al. [14] found an insignificant association between CRP concentration and air particles exposure among photocopier workers. This could probably be due to low concentration of particulate pollutants exposed, which did not really cause injury in the subjects' respiratory systems. However, since some other biomarkers in the study were found to be positively associated with concentration of air particles, the insignificance of CRP concentration is thought to be caused by its inhibitors.

Very limited studies have looked at the association between adhesion molecules and air pollutants. Some of these studies have shown elevated expression of ICAM-1 upon exposure to air pollutants [49], whereas some did not yield any significant result [50]. Many cell types express this cell adhesion protein molecule, which indicates inflammatory responses. The photocopier-exposed subjects studied by Elango et al. [14] showed a significant increase of plasma ICAM-1, which were probably due to repetitive exposure to photocopiers emissions. The upregulation of ICAM-1 gene expression imitates the inflammatory response induced by air particles.

Albeit commonly studied among asthmatics and rhinitis patients [51–53], ECP is scantily studied for its application as a biomarker of eosinophilic inflammation due to air particles exposure. This basic granule of protein is transported from eosinophil upon activation of inflammatory responses and has both cytotoxic and noncytotoxic effects [54]. Elango et al. [14] have provided evidence of inflammation due to exposure to particulate pollutants by demonstrating elevated level of ECP in their subjects. That is a sign of the involvement of the eosinophil in the inflammatory responses.

The realm of eNO has shown its positive association with levels of macrophage ROS generation [38]. This free-radical gas is detectable in exhaled air of human with multiple biological functions [55]. It is mostly known as an airway inflammation biomarker in asthma, mainly due to its positive association with asthma symptoms. However, Zhang et al. [16] found an insignificant association between eNO levels and air particle exposure among subjects. This result is mystifying because asthmatics have shown positive association with PM_2.5_ exposure [55]. Apparently Zhang et al. [16] only did two measurements of eNO concentrations; thus they might have missed the inflammation during asthma exacerbation.

MPO has been used in studies to assess the degree of inflammation occurring. It is released by degranulation of activated neutrophils [56] and its activity increases after particulates exposure [2]. Zhang et al. [16] observed a positive correlation between the air particles levels and MPO levels, which is in line with findings from some other studies [57, 58]. A high MPO level indicates a huge proinflammatory impact due to exposure to particulates. Nevertheless, it should be noted that high expression of MPO is possibly detrimental.

*Biomarkers of Mediator following Inflammation and Oxidative Damage.* Along with using biomarkers to assess oxidative damage and inflammation, this section is discussing the roles of CC16, a natural protective mediator in the airway that protects the respiratory tract from unwanted inflammatory reactions [59]. Vattanasit et al. [11] and Elango et al. [14] did not observe any significant correlation between particulate matter exposure and expression of CC16 for their studies. Apparently, the exposure of particulate matter to the subjects brought no significant effects to their lungs. Its levels in serum increase when air pollutants harmfully affect lung epithelium permeability [14]. In contrast, depletion of Clara cells may cause depletion in the levels of CC16 expressed in lung lavage fluid, thus resulting in a higher susceptibility to respiratory inflammation.

Another mediator is LTB_4_, which is a proinflammatory mediator and a lipid chemoattractant for neutrophils derived from arachidonic acid [60]. Despite plentiful studies to discover the role of LTB_4_ in inflammatory responses involving asthmatic patients [61] and in causing lung injury [62], very few studies have studied both leukotriene and air pollutants levels in one study. A variety of stimuli enhance LTB_4_ production. The significant increase in LTB_4_ levels of photocopier operators found by Elango et al. [14] signified that inflammation was occurring, and it is probably influenced by cascading oxidative stress. This view is supported by the elevated expression of TBARS, ICAM-1, ECP, and IL-8 in the study.

## 4. Conclusion

The reviews have provided a brief compilation of explanation for associations observed between air particles concentration and health effects. From studies reviewed in this paper, some biomarkers have shown a positive association with exposure to air particles, whereas some biomarkers have failed to show a positive association with exposure to air particles.

Most of the biomarkers mentioned in the studied literature have shown the connection between exposure to air particles and respiratory health effects. However, there are still ways of improvements for these biomarkers, those that need more clarification in their roles to link air particles and respiratory health. The fact that each of the biomarkers has its own inflammatory properties makes it difficult to sort out their relative characteristics. These biomarkers are either proinflammatory, anti-inflammatory, or both.

A limitation of this review is the inability to clearly specify and elaborate biomarkers individually by outlining the prime role of the particle chemical composition due to irregularity in the evidence found across epidemiological studies or insufficient information from experimental studies with respect to mechanisms that are biologically credible. This paper is providing essential information for future researches to be done in epidemiological studies of assessments of air particles exposure by the application of biomarkers.

It is noted that the clarification for the connection between concentration of air particles and health effects has been presented by several epidemiological and experimental studies, which have linked the particles composition with associated health effects. Nevertheless, the findings are not gathered in a piece of literature pertinent to application of biomarkers in estimating the magnitude of exposure to the air particles.

To shed light on this issue, future researches should be commissioned to further evaluate the sensitivity and specificity of biomarkers of interest individually for their suitability to be used in the epidemiological studies. Each biomarker is a unique entity of its own; thus additional research on biomarkers individually may enable the description of specific size fractions of particulate matter or components that affect the human health.

## Figures and Tables

**Table 1:** Studies on exposure to air particles by using biomarkers.

Air particles	Biomarkers studied	Findings	Subjects	Health effects	Authors
PM_2.5_	8-OHdG, IL-8, and CC16	Personal exposure to PM_2.5_ leads to oxidative DNA damage	18- to 58-year-old (mean is 45.2) healthy nonsmoker residents	An increased lung damage and cancer risk	[11]

PM_10_ and PM_2.5_	TNF-*α*, IL-6, IL-12p40, and IL-10	PM_2.5_ alters the balance between proinflammatory TNF-*α* and anti-inflammatory IL-10 production	18-year-old healthy adolescents from a highly industrialized city, a moderately industrialized city, and a rural town	Aberrant and dysregulation of immune status	[12]

PM_10_ and PM_2.5_	IL-6	PM_10_ and PM_2.5_ increase expression and secretion of IL-6	8- and 10-year-old urban school children versus rural school children	Cough, phlegm, wheezing, and chest tightness	[13]

PM_10_ and PM_2.5_	FRAC, TBARS, CRP, ICAM-1, LTB_4_, CC16, ECP, and IL-8	Long-term personal exposure to emissions from photocopiers is associated with inflammation and high oxidative stress	20- to 60-year-old (mean is 32.6) photocopier operators versus 20- to 60-year-old (mean is 31.7) healthy controls	Nose irritation, throat pain, nasal blockage, cough, excessive sputum production, wheezing, allergies, and breathing troubles	[14]

PM_10_, PM_2.5_, and UFP	IL-6 and TNF-*α*	Personal exposure to PM_10_, PM_2.5_, and UFP increases concentration of IL-6, whereas personal exposure to PM_2.5 _and UFP contributes to elevated concentration of TNF-*α*	20- to 55-year-old (mean is 38.3) bus drivers versus 20- to 55-year-old (mean is 36.64) administrative staffs	High risk in getting respiratory and lung diseases	[15]

PM_2.5_ and UFP	eNO, pH of EBC, TBARS, IL-8, and MPO	Short-term exposure to PM_2.5_ and UFP increases oxidative stress burdens in asthmatic patients	18- to 55-year-old nonsmokers with mild asthma (mean is 30.5) or moderate asthma (mean is 34.4)	Shortness of breath, chest tightness, wheezing, and cough	[16]

PM_2.5_	8-oxodG	Personal exposure to PM_2.5_ at modest levelsinduces oxidative DNA damage	20- to 33-year-old (median is 24) nonsmoker students	An increased risk of lung cancer	[17]

8-OHdG: 8-hydroxy-deoxyguanosine, IL: interleukin, CC16: Clara cell protein, TNF-*α*: tumor necrosis factor-*α*, FRAC: ferric reducing antioxidant capacity, TBARS: thiobarbituric acid reactive substances, CRP: C-reactive protein, ICAM-1: intercellular adhesion molecule 1, LTB_4_: leukotriene B_4_, ECP: eosinophil cationic protein, eNO: exhaled nitric oxide, EBC: exhaled breath condensate, MPO: myeloperoxidase, and 8-oxodG: 7-hydro-8-oxo-2′-deoxyguanosine.
